# Around the Hospitals

**Published:** 1894-01-20

**Authors:** 


### AROUND THE HOSPITALS.

Notice to the Managers of Institutions.—Special space is now reserved for the insertion of notices of hospital meetings and festivals. The Editor requests that all notices may reach The Hospital Office, 428, Strand, at least one week before the date of each meeting.

**The Royal Edinburgh Hospital for Sick Children.**—This hospital, which is situated at Morningside, has received 523 patients during the year. Out-patients numbered 5,976. The total income amounted to £4,984, a slight increase on that received in the previous year. The ordinary expenditure was £4,863, leaving a slight balance in hand. The hospital has had a very fair share of legacies, amounting to £2,038; and various societies and friends have shown their interest in the institution by holding entertainments on its behalf, and making collections for the same purpose. The hospital on its new site it is hoped will receive the same liberal support. The ground it is to occupy, consisting of two acres, cost the large sum of £5,000, and the value of the present site as well. It is in every way suitable, and the price has been defrayed through Lady Jane Dundas's munificent gift of £5,000 for the purpose. She has supplemented this donation by £1,500 to meet the additional cost of the endowment of a wing dedicated to her sister's memory, on the new site. It is anticipated that the money required for the erection will not be less than £30,000, but a large part of this sum has already been subscribed. In connection with the hospital is the ladies' work guild, which provides clothes for the patients. Such a society might easily be formed in connection with every children's hospital, for garments for children are easily and pleasant work, and for such sympathetic workers are always forthcoming with a small amount of energy on the part of an organizer. Ladies have a great share in the work of the Edinburgh Children's Hospital, and no doubt this is in part due to the welcome visitors receive from the hospital staff.

**The French Hospital.**—This hospital, although French in both denomination and organization, is also cosmopolitan in the nature of its patients, no less than eighteen different nationalities benefiting by its treatment during the past year. The total number of patients received amounted to 652 last year, during which time the hospital was closed for two months for repairs, consisting principally of relaying of the floors and alterations to the corridors. These improvements have been provided out of the ordinary income of the hospital, which has been so well supported as to make this extra expenditure possible, legacies having been the chief factors in the case. Many foreign governments and members of various royal families help to support this hospital which freely opens its doors to all comers. The City of Paris sends a yearly subscription of £60, and many French towns send contributions of varying amount. Amongst the names of donors figures that of Sarah Bernhardt for the sum of £32. The building fund is still in need of further contributions, the cost resulting from the erection of new buildings not being yet paid off.

